# Lockers

**Published:** 1893-03-04

**Authors:** 


### PRACTICAL DEPARTMENTS.

### LOCKERS.

A very important piece of ward furniture is the locker.

Messrs. Atkinson and Co., Westminster Bridge Road, who make a speciality of all hospital furniture, have many varieties of this indispensable article to show, from those of the simplest construction to others of a more elaborate type. Some of the newest improvements are wonderfully ingenious, and will surely be welcomed by hospital authorities.

A capital idea has been carried out by this firm, that of fitting the top of a locker, which is high enough to be used as a table, with a thick sheet of plate glass, thereby preventing any staining of the polished surface of the wood by the spilling of food or medicine. The glass has a bevelled edge in front, and fits tightly to the narrow rim of wood which runs round the other three sides. The advantage of this plan, with regard to its superior cleanliness, is undoubted.

Messrs. Atkinson have manufactured some 300 of these glass-topped lockers, and we have no doubt they will be largely adopted in hospital wards.

There are many little points to be kept in mind when choosing lockers convenient both for patients and nurses. First and foremost comes the necessity for ventilation. In hospitals where the patients keep their tea, sugar, and other commodities in their lockers, this question is of considerable importance. Messrs. Atkinson's plan is to construct the back of their lockers with lathes, just sufficiently far apart to freely admit the air.

Another important point is that lockers should be raised from the floor, preferably by castors, in order to prevent any accumulation of dust.

Some are made low enough to be used as seats, and this is a good plan, when they are not so low as to make it too great an effort for the patient to reach his cup and saucer, &c., for lockers are necessarily often used as tables.

One of the more elaborate kinds is constructed on a good principle. It is high, divided horizontally, the lower division forming a cupboard, with a door opening in front, whilst the upper part has the opening at the side next the bed. There is no door to this upper compartment, which is very handy for the keeping of the patient's special possessions, while the fact of the opening being at the side prevents any untidy appearance. This locker has a shelf at the top, and a towel rail can be fixed at the back if desired.

This same principle is carried out in another more simply made locker, which is intended to serve between two beds should space be limited. It is divided into two compartments, an upper and a lower, the openings being at opposite sides, so that there need be no confusion between the belonging of the occupants of the two beds.

Messrs. Atkinson have adopted yet another useful plan, which combines two distinct advantages. We have just alluded to the convenience of a locker being sufficiently low to serve as a seat, while at the same time a patient will often be glad to use it as a table. Messrs. Atkinson have surmounted the difficulty by making a locker to answer both purposes, in the following manner: The lower part is simply an ordinary box locker, but with a high back fitted with a hinged flap, to be raised or lowered at will. When raised it is the right height for a very convenient table, and when down it merely forms part of the back of the chair. All these are made in polished ash or walnut, or any wood that may be preferred.

Messrs. Atkinson, from making a special department of furniture for hospital use, and being able thus to bring out the latest improvements, can provide the most suitable ward fittings at most reasonable prices.

Messrs. Stidolph, Dartford, have also many excellent samples of lockers to show, some of which we cannot pass over without a few words of comment.

One of these is supplied with a revolving book tray and bed table, fitted to the back of the locker, which itself is just the right height for a seat. When the table is not required, it folds, and slides down behind the locker; this being on castors, is easily placed in either position.

Another specimen of the ordinary box locker is made with a high back, something after the manner of one we have described above; the lid of the seat is made to lift up, there being divisions inside. The back is fitted with a try, newspaper rack, and towel rail.

We should not omit to mention one other locker, made in cupboard fashion. The large lower cupboard, which is divided horizontally, has a second smaller one over it, leaving room enough on each side for cup or plate, &c., whilst above again we have tray and towel rail.

Other kinds are provided with drawers, in addition to cupboards, and indeed, in short, every convenience of patient and nurse has evidently been carefully considered by both firms mentioned in the construction of these hospital necessaries.

